# Serum and supplement optimization for EU GMP-compliance in cardiospheres cell culture

**DOI:** 10.1111/jcmm.12210

**Published:** 2014-01-20

**Authors:** Isotta Chimenti, Roberto Gaetani, Elvira Forte, Francesco Angelini, Elena De Falco, Giuseppe Biondi Zoccai, Elisa Messina, Giacomo Frati, Alessandro Giacomello

**Affiliations:** a Department of Medical Surgical Sciences and Biotechnology, “Sapienza” University of RomeLatina, Italy; b Department of Molecular Medicine, Pasteur Institute – Cenci Bolognetti Foundation, “Sapienza” University of RomeRome, Italy; c IRCCS NeuromedPozzilli, Italy

**Keywords:** adult stem cells, cardiac cell therapy, cardiospheres, Good Manufacturing Practice compliance, human sera

## Abstract

Cardiac progenitor cells (CPCs) isolated as cardiospheres (CSs) and CS-derived cells (CDCs) are a promising tool for cardiac cell therapy in heart failure patients, having CDCs already been used in a phase I/II clinical trial. Culture standardization according to Good Manufacturing Practices (GMPs) is a mandatory step for clinical translation. One of the main issues raised is the use of xenogenic additives (*e.g*. FBS, foetal bovine serum) in cell culture media, which carries the risk of contamination with infectious viral/prion agents, and the possible induction of immunizing effects in the final recipient. In this study, B27 supplement and sera requirements to comply with European GMPs were investigated in CSs and CDCs cultures, in terms of process yield/efficiency and final cell product gene expression levels, as well as phenotype. B27− free CS cultures produced a significantly reduced yield and a 10-fold drop in c-kit expression levels *versus* B27+ media. Moreover, autologous human serum (aHS) and two different commercially available GMP AB HSs were compared with standard research-grade FBS. CPCs from all HSs explants had reduced growth rate, assumed a senescent-like morphology with time in culture, and/or displayed a significant shift towards the endothelial phenotype. Among three different GMP gamma-irradiated FBSs (giFBSs) tested, two provided unsatisfactory cell yields, while one performed optimally, in terms of CPCs yield/phenotype. In conclusion, the use of HSs for the isolation and expansion of CSs/CDCs has to be excluded because of altered proliferation and/or commitment, while media supplemented with B27 and the selected giFBS allows successful EU GMP-complying CPCs culture.

## Introduction

During the last decade, fast-paced advancements in regenerative medicine are offering novel promising therapeutic tools such as cardiac cell therapy, which is emerging as an effective personalized treatment for end-stage heart failure (HF) patients 1–4. Considering the social and financial cost of HF in western countries and its increasing incidence as a chronic and epidemic condition, there is great interest in developing new effective approaches to overcome the final pathological mechanism, which is the loss of contractile tissue as a result of cardiomyocyte death. To achieve successful regeneration, cell sources are needed with indisputable cardiomyogenic differentiation potential and resident cardiac progenitor cells (CPCs), which have been tested in many animal models, and recently also in clinical settings, seem to have the most promising potential 5–8. Three clinical trials for cardiac regeneration by resident CPCs have been started so far: CADUCEUS (NCT00893360) 9, SCIPIO (NCT00474461) 10 and ALCADIA (see NCT00981006 on http://www.clinicaltrial.gov) 11, representing a solid proof of concept in terms of feasibility, safety, reduction in infarct scar, therapeutic regeneration and functional recovery.

Clinical translation requires protocol optimization in compliance with current Good Manufacturing Practices (cGMPs) for cell preparation. Despite shared international guidelines, national regulations (according to European Directives for EU countries, 2007/1394/EC) require testing, strictly proving the necessity of the proposed protocols and reagents. One of the most important issues involved is the use of supplements of xenogeneic origin for human cell culture 12. Thus, careful evaluation of the minimal reagents requirements to grant the expected cell yield and phenotype needs to be performed. Foetal bovine serum (FBS) represents the most commonly used cell culture supplement to provide the vital nutrients, toxin scavengers and growth factors necessary to maintain mammalian cells *in vitro*. Nevertheless, lot variability, the risk of contamination with infectious viral or prion agents, and the possible induction of immunizing effects in the final recipient require the investigation of possible replacements for clinical-grade preparations 13–15. For many cell types (*e.g*. mesenchymal stem cells, endothelial progenitors, adipose-derived stromal cells), other supplements, such as human AB or cord blood serum and platelet lysate (PL), have been shown to be valid FBS substitutes 16–19. Nevertheless, effective alternatives with the same wide acceptability are still missing, and it is very often impossible to exclude its use without significant changes in cell culture efficiency 12. In this case, all possible precautions must be implemented, in terms of FBS origin, preparation and precautionary pathogen inactivation (see EMA ‘Note for Guidance on the Use of Bovine Serum in the Manufacture of Human Medicinal Products’ at http://www.ema.europa.eu). The same infective and immunogenic issues and risks obviously exist for any other xenogenic and/or artificial media additive, which has to be GMP compliant.

One of the CPC populations tested so far for cardiac cell therapy (CADUCEUS trial) is derived through the cardiosphere (CS) protocol 20,21. CSs are spontaneous 3D structures, housing undifferentiated proliferating cells in their core and differentiating progenitors of cardiovascular lineages in their periphery. They resemble a niche-like microenvironment 22,23 inducing muscle specification 24 and forming through an epithelial-to-mesenchymal transition (EMT)-mediated process 25, where CPCs coexist with mesenchymal supporting cells. Cardiosphere-derived cells (CDCs) 26 can be efficiently expanded as monolayers, and are able to form secondary CSs (IICSs) when plated again in the appropriate conditions 21. Cardiac progenitor cells in the form of both CDCs and IICSs have been tested in many animal models of cardiac cell therapy for HF 7, and CDC also in a clinical trial 9. Both CPC forms exert therapeutic effects, although multiple lines of evidence suggest that the three-dimensional form (*i.e*. CSs and IICSs) is more efficient 22,27.

The original formulation of the CS protocol includes FBS, B27 supplement and recombinant growth factors at various steps of the culture process. For some components, an important role in CSs and CS-derived cells (CDCs) 26 phenotype and function has been already recognized, that is thrombin 28 and EGF 29. For FBS and B27 supplement, though, a thorough assessment of their possible replacement in media recipes has not been performed yet. In this study, we first investigated the effect of B27− free culture on CSs yield and phenotype. Then, we tested multiple serum alternatives to standard research-grade FBS, to find a possible GMP-grade replacement in compliance with European directives and standards. We analysed each step of the culture protocol (or until possible) to verify that the GMP-complying formulations tested would allow the same outcome (yield, phenotype) as the consolidated research-grade protocols.

## Materials and methods

### Cell culture

Human CPCs were isolated through the biopsy-derived explant method, as previously described 21. Surgical auricola biopsies were obtained from 19 different patients (myocardial revascularization or aortic/mitral valve repair/replacement; Table S1) during clinically indicated procedures after informed consent, in an institutional review board–approved protocol. Cardiospheres were grown on poly-D-Lysine (BD Biosciences, Franklin Lakes, NJ, USA)–coated plates in CS-growth medium (CGM): 35% IMDM and 65% DMEM/F-12 Mix, 3.5% serum, 1% penicillin-streptomycin, 1% L-glutamine, 0.1 mM 2-mercaptoethanol, 1 U/ml thrombin (Sigma-Aldrich, St. Louis, MO, USA), 1:50 B-27 (Invitrogen, Carlsbad, CA, USA), 80 ng/ml bFGF, 25 ng/ml EGF and 4 ng/ml cardiotrophin-1 (Peprotech, Rocky Hill, NJ, USA). Primary explants and CDCs were cultured on fibronectin (Sigma-Aldrich)-coated flasks in complete explant medium (CEM): IMDM, 1% penicillin-streptomycin, 1% L-glutamine, 0.1 mM 2-mercaptoethanol, serum as specified in each data set. IICSs were obtained by re-plating CDCs in CGM on poly-D-lysine–coated multi-wells at a density of 1 × 10^4^ cells/cm^2^. For each biopsy, tissue was equally divided into the different conditions tested, always maintaining a culture with standard FBS conditions as a positive biological control and reference. The different commercial sera tested are as follows: research-grade FBS (Lonza, Basel, Switzerland), GMP-grade human AB sera (Starfish; Lonza), gamma-irradiated GMP-grade FBSs of Australian origin (Lonza; Gibco, Carlsbad, CA, USA; Hyclone, Waltham, MA, USA). For human sera, in some experiments, one aliquot was subjected to heat inactivation (56°C for 30 min.). For the B27 set of experiments, outgrowth cells from the same explants in research-grade FBS (Lonza) were seeded in CGM with or without B27; both B27+/− CDCs were cultured in CEM, and then corresponding IICSs were obtained plating again in B27+/− CGM.

### Human serum preparation

Autologous serum was obtained by standard procedures of blood centrifugation and clotting, with an approximate volume yield of 50%.

### Cell proliferation and yield

Proliferation was quantified in a time-course analysis by WST8 incubation (Cell Counting Kit; Alexis Biochemicals, San Diego, CA, USA) and absorbance reading, according to the manufacturer's instructions; signal was normalized to that of day 0 after cell seeding (1000 cells per well). Based on reference standard curves (data not shown), in our setting, an approximate 2.6-fold increase in normalized signal corresponds to a population doubling. CSs yield was calculated 1 week after seeding by semi-automatic particle count performed by ImageJ software on at least 8 random fields per condition per cell line, normalized to each corresponding standard FBS culture, and finally averaged among biological replicates. CSs dimension was calculated by ImageJ measurements of the average diameter on at least 20 random CSs per condition per cell line, normalized to each corresponding standard FBS culture, with final averaging among biological replicates.

### RNA extraction and realtime PCR

RNA was extracted with column-based kits (Qiagen, Venlo, the Netherlands). Reverse transcription was performed on 500 ng starting RNA (Qiagen) in a 20-μl reaction, and 1 μl of cDNA product was then subjected to realtime PCR with Sybr Green Supermix in a MiniOpticon instrument equipped with CFX software (Bio-Rad, Hercules, CA, USA) for 40 thermal cycles (95°C for 10 sec., 56/58°C for 10 sec., 72°C for 30 sec.; see Table S2 for primers sequence and annealing temperatures). All primers sets were previously tested for optimal efficiency and all reactions were analysed by melting curves at the end to confirm product specificity. Each reaction was performed in triplicate. The ΔΔCt method was used for relative quantification, using GAPDH as the housekeeping gene 30, and the expression levels of cells in standard culture conditions (B27+ or FBS) for each biological replicate as the reference for normalization.

### Immunofluorescence

Cells were fixed for 10 minutes with 4% paraformaldehyde at 4°C, permeabilized with 0.1% Triton X-100 (Sigma-Aldrich) in PBS with 1% BSA, then blocked in 10% goat serum, incubated overnight at 4°C in 1% goat serum with primary antibodies and then incubated for 2 hrs at room temperature with secondary antibodies and Topro3 nuclear dye (Invitrogen). Slides were mounted in Vectashield medium (Vector Laboratories, Burlingame, CA, USA) and confocal fluorescence imaging was performed on a TCS DMIRE 2 Leica microscope, equipped with LCS Lite Software (Leica, Solms, Germany). Incubation with secondary antibodies alone did not give any detectable background signal.

### Statistical analysis

All results are presented as mean value ± SEM, unless specified. Significance of difference between any two groups was determined by two-sided Student's *t-*test, and a final value of *P* < 0.05 was considered significant.

## Results

### B27 supplement

**Figure 1:**
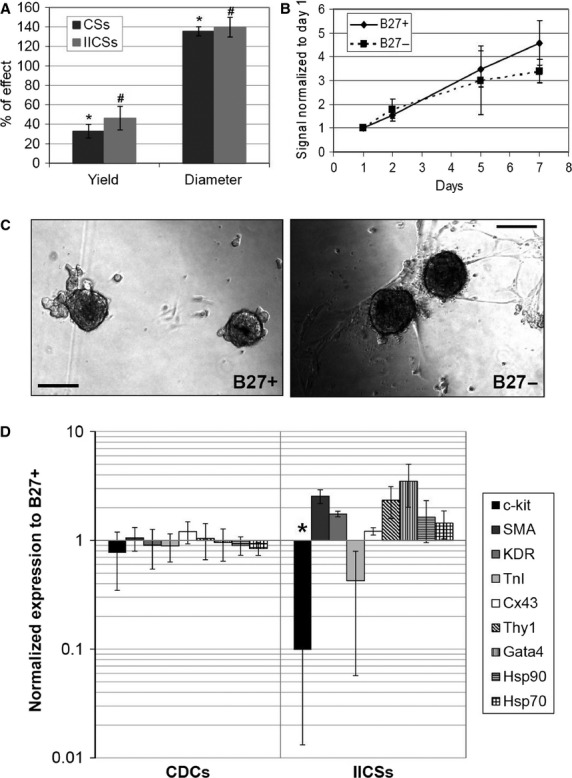
B27 supplement is required to maintain standard cardiospheres (CSs) culture yield and gene expression profile. Primary and secondary CSs yield and dimension (A) were altered in B27− CGM, expressed as percentage of effect, normalized to B27+ CGM (*n* = 3). Cell proliferation, quantified by WST colorimetric assay, of CS-derived cells (CDCs) from both B27+/− was comparable (B). Representative images of B27+/− CSs are shown in C. Gene expression analysis by realtime PCR for multiple markers of stemness and cardiovascular differentiation (D) revealed no differences in the profile of B27+/− CDCs, while a dramatic drop in c-kit levels in B27− IICSs was detectable (*n* = 3 each). The marked size of error bars for c-kit and TnI in (D) is due to the fact that in one replicate, expression levels were undetectable. #*P* < 0.05 *versus* B27+. *P < 0.001 *versus* B27+. Scale bars = 100 μm.

Explant-derived cells (EDCs) from three different patients were plated in CGM with or without B27, and CSs were obtained. CDCs from both B27+/− were cultured in CEM, and then IICSs were obtained again in the corresponding B27+/− CGM. Primary and secondary CSs yields in B27− compared with B27+ were significantly reduced to 32.8 ± 6.9% and 46.2 ± 12.1% respectively (Fig. 1A), whereas the average diameter of the spheres after 7 days of culture was slightly higher in B27− samples: 135.5 ± 4.6% in CSs and 139.8 ± 10.1% in IICSs (Fig. 1A). Representative bright field images of the cultures are shown in Figure 1C. CDCs obtained from both culture conditions displayed similar growth rate (Fig. 1B), despite the absolute yield being obviously lower for B27−. Then, we analysed the gene expression profile of CDCs and IICSs from B27+/− cultures. Realtime PCR results are shown in Figure 1D. The expression levels of the genes analysed, normalized to the levels of B27+ cultures, were unchanged in CDCs, suggesting no significant phenotypic shift maintained after the primary CS stage. Conversely, trends in genes modulation were detectable in IICSs derived from B27− CSs and cultured again without B27, with a 10-fold dramatic drop in c-kit expression levels (*P* < 0.001), suggesting a reduction in the stemness potential of IICSs. Analysis of Hsps expression levels suggests no increase in cell stress. Thus, despite no memory of previous B27 starvation seems to be detectable in CDCs at the mRNA level for the markers analysed, B27 removal at IICSs stage significantly affects cell yield and their typical gene expression profile.

### Autologous human serum

**Figure 2:**
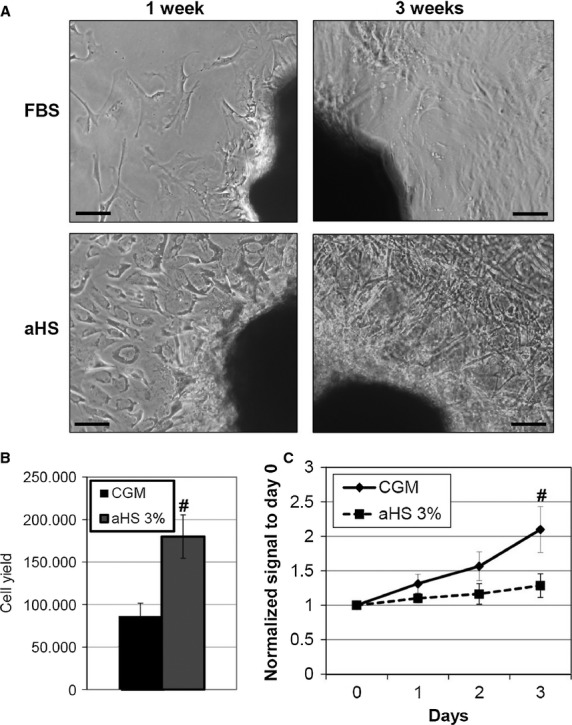
Autologous serum is not able to support cardiospheres (CSs) formation after primary explant. Representative images of explant cultures show rapid initial growth in autologous human serum (aHS) compared with foetal bovine serum (A), and higher cell yield (*n* = 5) in terms of harvest of CS-forming cells (B). Once plated to form CSs, though, cells in aHS stopped proliferating (C), as demonstrated by WST assay (*n* = 5). #*P* < 0.05. Scale bars = 50 μm.

Explants from five different patients were divided into equal tissue amounts and plated in two different CEM, one with normal FBS and the other supplemented with 20% autologous human serum (aHS). Primary cultures with aHS grew much quicker than FBS during the first 3 weeks (Fig. 2A), and the average EDCs yield per harvest was significantly higher (Fig. 2B). Despite this, once plated to form CSs in CGM supplemented with the corresponding serum (FBS or aHS), cells from aHS explants stopped growing, as shown by the proliferation curve (Fig. 2C), without yielding any significant amount of cells for further experiments.

### Commercial human AB serums

**Figure 3:**
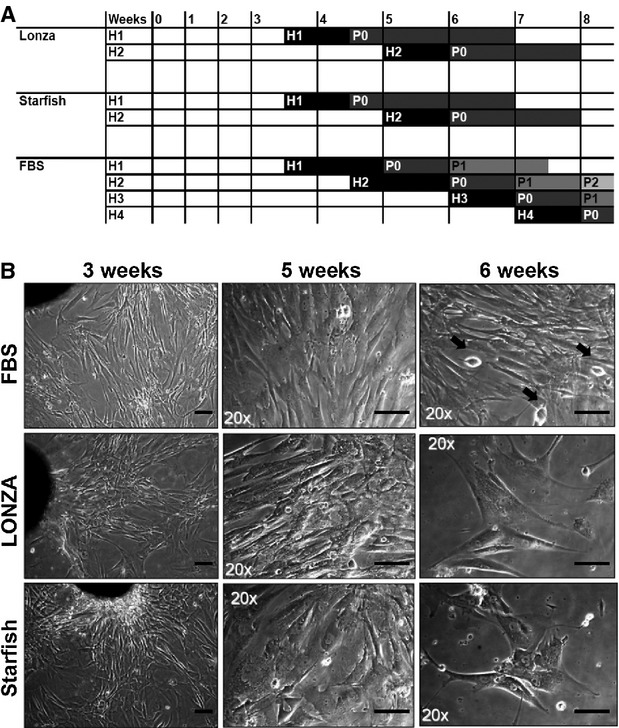
Cultures with commercial AB human sera gradually displayed senescence features. Initial cell growth in primary explants was comparable between foetal bovine serum (FBS) and HSs, as demonstrated by time-course of cell harvests (H) up to H2 (A) and similar morphology in culture (B, left column). Nevertheless, after 5–6 weeks, HS outgrowth cells gradually ceased proliferating, preventing further harvests (A) and assuming a flat vacuole-rich morphology (B), whereas in control explants, round phase-bright proliferating cells could still be detectable (black arrows), reaching H4 (A). Scale bars = 50 μm. H: harvest of cardiosphere-forming cells from primary explant culture. P: passage of CS-derived cells.

**Figure 4:**
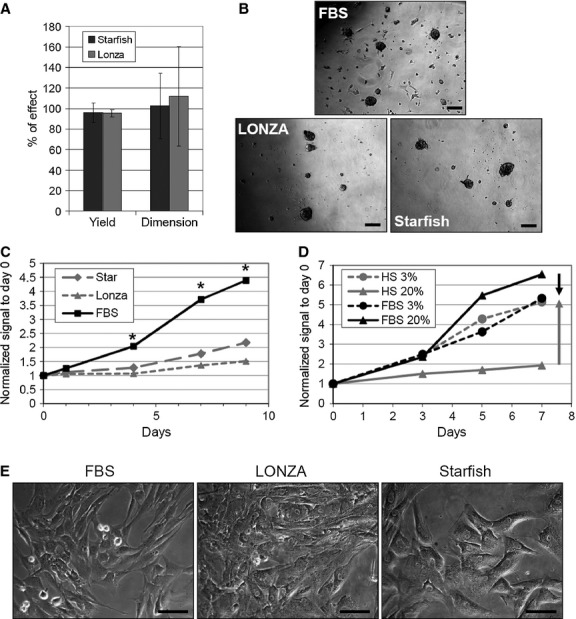
Cardiospheres (CSs) yield and cell proliferation in AB human sera cultures. CSs yield and dimension, expressed as percentage of effect *versus* foetal bovine serum (FBS), were comparable in human serum (HSs) cultures (A), until CS-forming cells could be harvested. Representative images of CSs from different sera are shown in B. Nevertheless, at the CS-derived cell (CDC) stage cells stopped proliferating compared with control cultures in FBS (C), and such effect was dose dependent, as demonstrated by comparing cultures in 3% and 20% sera (D). Representative images of CDCs in HSs and FBS are shown in E. Scale bars = 50 μm. *P < 0.001 *versus* HSs.

Next, we examined the replacement of FBS with commercially available standardized AB human sera. We tested two products from two different companies on explants from three different patients. Figure 3A shows a representative comparative timeline of cultures in the two HSs and FBS as control. As observed for aHS (Fig. 2A, left panels), the explants in commercial HSs seemed to grow quicker, or at least in parallel, compared with standard CEM (Fig. 3B, Table S3). Nevertheless, after 4–5 weeks of primary culture and after one to two harvests of EDCs, outgrowth cells in HS explants gradually changed their morphology (Fig. 3B, right panels), increased cell size, took a flat vacuole-rich shape, and stopped proliferating and migrating, preventing further harvests from the primary culture. On the contrary, in standard FBS, explants outgrowth cells kept their original morphology, and bright round proliferating cells could still be detected at 6 weeks of culture, allowing up to four harvests from the same explant. As demonstrated in the timeline (Fig. 3A), explant in FBS was harvested approximately every week up to four times, whereas explants in HS could not be harvested anymore after the fifth week. The average time necessary for the first harvest, the time between one harvest and the following, and the average number of harvests possible from the same primary explant are summarized in Table S3. Average yield and dimension of CSs obtained from HS explants, and grown in CGM with HS, were comparable to those from FBS (Fig. 4A), despite significant variability among patients. Representative images of CSs cultures in different conditions are shown in Figure 4B. When plated as CDCs, HS cells displayed a significantly reduced proliferation rate compared with FBS (Fig. 4C). Moreover, even considering the best performing HS (*i.e*. Starfish), comparative growth curves in 3% or 20% serum showed a clear dose-dependent inhibitory effect of HS on CDCs proliferation (Fig. 4D). In fact, lowering FBS from 20% to 3% decreased proliferation, as expected, while for HS, an opposite trend was observed, with a significantly higher proliferation rate at 3% *versus* 20%. Reduced proliferation of CDCs in HS was consistent with the observed morphology (Fig. 4E): as seen in primary explant cultures, CDCs in HSs assumed a senescent-like shape and eventually stopped proliferating at early passage.

**Figure 5:**
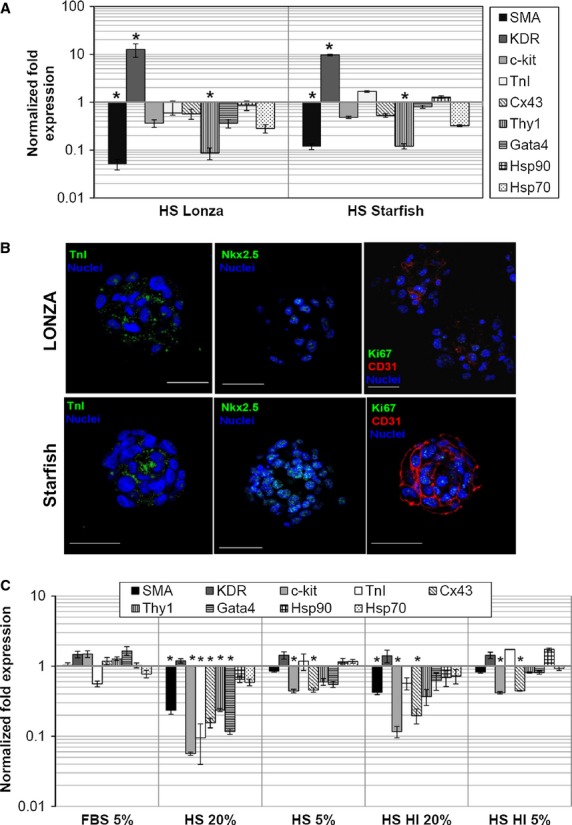
Cardiac progenitor cells in AB human sera displayed altered commitment towards cardiovascular lineages. Gene expression analysis on CS-derived cells (CDCs; A), normalized to standard foetal bovine serum (FBS) conditions, revealed a significant up-regulation of KDR in human sera (HSs), and a significant down-regulation of SMA and Thy1, suggesting a preferred commitment towards an endothelial lineage. Immunofluorescence and confocal analysis of primary cardiospheres (B) further evidenced a higher expression of the endothelial marker CD31 in HSs, particularly in Starfish samples. CDCs obtained from standard FBS were cultured for 1 week in HS 5% or 20%, subjected or not to heat inactivation (HI). Gene expression analysis (C) revealed that 1 week of HS exposure was enough to significantly down-regulate multiple genes *versus* FBS, such as SMA, c-kit, TnI, Cx43, Thy1 and GATA4. As observed for cell proliferation, the effect was dose dependent. Heat inactivation partially recovered the effect, but important genes, such as SMA, c-kit and Cx43, were still significantly down-regulated compared with standard FBS conditions. Scale bars = 50 μm. *P < 0.001 *versus* FBS.

Gene expression analysis was performed on CDCs from HS cultures, and normalized to standard FBS (Fig. 5A). Smooth muscle actin (SMA) and Thy1 levels were significantly down-regulated in both HSs, while cardiac markers, such as TnI and Cx43, were basically unaffected. Analysis of Hsps expression levels suggests no changes in cell stress. Interestingly, KDR was dramatically up-regulated in both HSs, suggesting that HSs could encourage endothelial commitment of CDCs. This hypothesis was further supported by immunofluorescence analysis of CSs (Fig. 5B), which showed, especially for Starfish serum, a strong and homogeneous positivity for CD31. The expression of TnI and Nkx2.5 proteins was confirmed by immunofluorescence as well.

To test whether the HS negative effect could be reduced or avoided by decreasing serum concentration from the beginning of the protocol, an attempt was made to culture primary explants in 1% or 3% HS, but no cells could be obtained (Figure S1).

Moreover, to test whether residual complement activity could be responsible for the growth arrest and phenotype change observed, we tested Lonza HS after heat inactivation treatment. CDCs from normal FBS explants were plated for 7 days in FBS 5% as control, Lonza HS 20% or 5%, Lonza HS heat inactivated 20% or 5%, and gene expression analysis was performed by realtime PCR and normalized to standard FBS 20% conditions. As shown in Figure 5C, even on normal healthy CDCs, 1 week of culture in HS was enough to significantly modulate gene expression. In Lonza HS 20%, SMA, ckit, TnI, Cx43, Thy1 and Gata4 were significantly down-regulated, while KDR levels were unchanged, confirming again a possible preferential endothelial commitment exerted by HS. As observed for cell proliferation, a dose-dependent effect was detected, as demonstrated from the analysis of the Lonza HS 5% sample, where most genes inverted the down-regulation trend. Genes down-regulation was still detectable in heat-inactivated HS, displaying again an inverted dose-dependent trend from 20% to 5%. Nevertheless, important genes, such as SMA, c-kit and Cx43, were still significantly down-regulated compared with standard FBS conditions.

### Gamma-irradiated FBS

**Figure 6:**
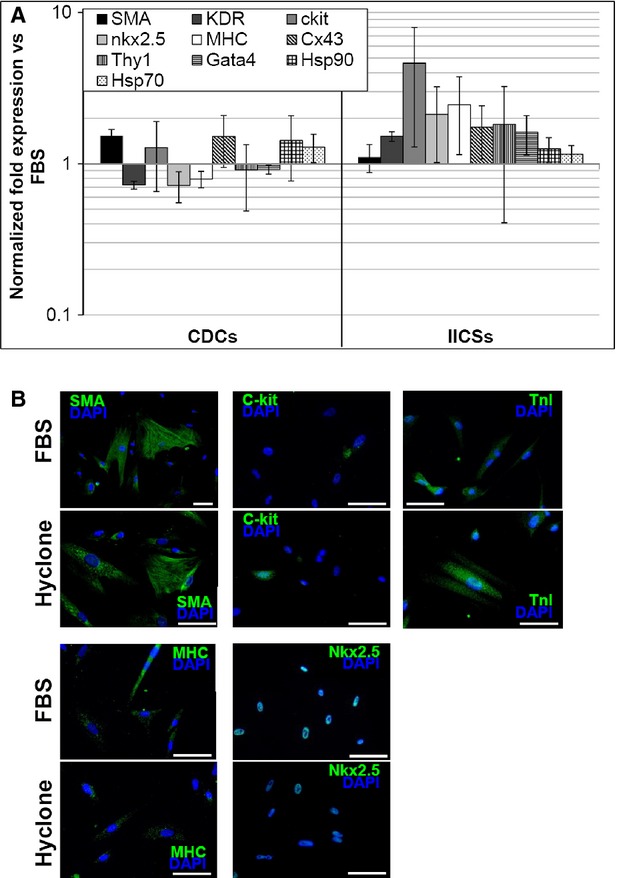
Gene expression profile of cardiac progenitor cells cultured with Hyclone giFBS. Realtime PCR analysis (A) detected expression levels very similar to control for the genes analysed in Hyclone giFBS cultures, with a trend towards up-regulation in IICSs for some markers, such as c-kit. (B) Representative immunofluorescence panels for SMA, c-kit, TnI, MHC and nkx2.5, showing similar abundance and antigen distribution in Hyclons giFBS compared to standard conditions. Scale bars = 20 μm.

As human sera exerted inhibitory/toxic effects on our cellular model and altered commitment, we next examined the possibility of using GMP gamma-irradiated FBS (giFBS) of Australian origin. We evaluated sera from three different companies, Lonza, Gibco and Hyclone, on eight different biopsies overall. Considering Gibco and Lonza, we were able to isolate CPCs from three out of five and two out of five explants, respectively, while all control explants in FBS yielded successful CPCs isolation. With these two sera, we observed again a trend of senescent-like morphology with time in culture (Figure S1A). Average CSs yield and dimension were not significantly different from standard FBS (Figure S1B), but the overall rate of successful explants was clearly unsatisfactory. Hyclone explants were all successful, with comparable timing (Table S3) and yield (Figure S1B) *versus* standard FBS, and did not display altered cell morphology with time in culture (Figure S1A). Average yield and dimension of CSs obtained from successful giFBS explants were comparable to those from standard FBS (Figure S1B). Proliferation of CDCs was significantly reduced in cultures with Lonza and Gibco sera (Figure S1C), while Hyclone giFBS performed as control. Thus, Hyclone giFBS can be considered the best performing GMP serum, as far as yield and cell proliferation are concerned. Then, we compared the phenotype and gene expression between Hyclone and control FBS. Realtime PCR analysis of Hyclone CDCs and IICSs revealed no statistically significant modulation in expression levels of the genes analysed (stemness, lineage and stress related) when normalized to control FBS, despite trends towards up-regulation in IICSs, particularly for c-kit (Fig. 6A). Immunofluorescence analysis (Fig. 6B) detected similar morphology and phenotype between the two sera, with the same abundance of positive cells and antigen intracellular distribution.

## Discussion

Cardiac cell therapy has moved very quickly towards clinical translation. Many clinical trials designed for the treatment of HF have been completed in the past 10 years with different adult stem cells populations (*e.g*. hematopoietic, mesenchymal and skeletal) 2,7,31, including recently resident autologous CPCs 9,10. Clinical translation requires compliance with GMP quality standards for cell production, and protocols adjustment may not always be straightforward. In particular, the use of FBS in media, as a xenogenic non-standardized ingredient, is still under discussion, being nevertheless widely used for the clinical-scale isolation, expansion and production of different stem cells. Despite multiple washes to remove FBS contaminants from the final cell product are routinely performed, nevertheless, the risk of adverse reaction in final recipients persists. Concerning cell populations of haematopoietic or bone marrow origin under clinical evaluation (*e.g*. endothelial progenitor cells, mesenchymal stem cells), many studies have been already performed, aiming at optimizing cell culture conditions 12,14,15,17,32. For example, the use of aHS with human T cells *in vitro* extends their proliferative lifespan, clonogenicity and telomerase activity 33. MSCs from different sources have been successfully cultured in HS 34, PL 18 or cord blood serum 16. Concerning resident CPCs, there is still no literature focused on comparing different culture conditions, media, supplements or growth factors.

We first examined the role of the B27 supplement in CSs culture (Fig. 1), comparing CSs, CDCs and IICSs obtained from B27+ or B27− CGM. B27 is a chemically defined, widely used ingredient first introduced in neurobiology research, and nowadays described in the formulation of media for multiple stem cell types, such as neuronal stem cells 35, neurosphere-derived cells 36, spermatogonial stem cells 37 and retinal stem cells 38. It contains vitamins, antioxidants, essential fatty acids and hormones (*e.g*. insulin, progesterone, T3); it is commercially available as GMP-grade and has been already used in clinical-scale cell productions 39–41.

B27 granted significantly higher CSs yield. CDC proliferation rate and gene expression levels seem not to have memory of previous B27 starvation, displaying no difference compared with CDCs from standard B27+ CSs. IICSs, though, showed again altered yield and morphology, and gene expression profile when cultured in B27− free CGM. The significant drop in c-kit expression level (Fig. 1D), suggesting a depletion in stemness properties or pool inside the CS, is of particular interest and not desirable, given the superior regenerative and therapeutic potential of CSs *versus* CDCs 22,27. Thus, our data suggest an important contribution of B27 to the distinctive phenotype of CSs and IICSs.

Next, we attempted replacing FBS with aHS (Fig. 2), but despite promising initial growth of primary explants and EDCs yield, early growth arrest at the CS stage in aHS cultures prevented successful culture completion. In other cell models for regenerative medicine, such as MSCs 42, human Schwann cells 43 or Tenon's fibroblasts 44, aHS outcome was comparable to FBS, but, based on our data, with CSs cultures it cannot be considered as a valid option.

To avoid the possible influence of patients' variability and/or because of their ‘pre-harvest’ clinical conditions, we tested commercial GMP-grade AB human sera from two companies: Lonza and Starfish. Despite similar initial growth of primary explants compared to standard FBS, EDCs from HSs acquired a senescent-like morphology (Fig. 3) with time in culture and stopped proliferating, significantly reducing the number of harvests and the cumulative yield from explants (Table S3), despite similar CS yield and dimension in the first possible harvests (Fig. 4). CDC proliferation was significantly impaired in both HSs, particularly in Lonza HS, and displayed a reverse dose–response trend for serum concentration (Fig. 4D), suggesting some kind of inhibitory and/or toxic effect. Gene expression profile showed a significant up-regulation of KDR and down-regulation of SMA and Thy1 in CDCs from HSs *versus* standard FBS (Fig. 5A), suggesting a preferential endothelial commitment. This was confirmed by immunofluorescence, and was particularly evident in Starfish HS, where a striking CD31 positivity in the whole CS was observed (Fig. 5B).

Successful stem cell cultures with AB HSs have been obtained with MSCs 34,45,46 or dental pulp–derived stem cells 47. Variable results with AB HSs from different companies have been observed for mesodermal progenitor cells 48, where interestingly a small concentration of HSs in media was sufficient to change their phenotype, whereas for Tenon's fibroblasts for corneal regenerative medicine AB HS failed to give successful cultures 44. Nevertheless, FBS has been also suggested to promote greater culture adaptation and therapeutic potential of adipose-derived MSCs 49, highlighting how variability in products and cell-type responsiveness are still open issues for sera optimization in clinical-grade productions. Overall, our results suggest that both autologous and AB commercial HSs are not suitable for CPCs isolation and culture with the CS method, as they are necessary in high concentration since the first culture stage (Figure S1), but seem to exert inhibitory/toxic effects at early stages of culture, significantly reducing cell yield, and to alter the spontaneous functional balance between vascular and cardiac commitment in CSs and CDCs.

To try all possible alternatives to FBS, we tested some commercially available serum-free formulation and PL as well, which have been also described in other stem cell models 12,19,50–52, without any success in primary explant culture or CS viability (data not shown). Interestingly, sequential use of PL and HS has been shown effective for cardiovascular tissue engineering approaches on collagen scaffolds 53, but, despite CS being suitable for tissue engineering approaches as well 54,55, our results suggest that this kind of media formulation would not be effective with this cell model.

As xenogenic FBS cannot be replaced with human-derived supplements in our culture system, we tested three different giFBSs. Gamma-irradiation is the recommended virus-inactivation treatment for FBS by EMA guidelines, while the Australian origin secures from the risk of contamination from any major epidemic disease. Lonza and Gibco giFBSs not always allowed successful CSs and CDCs cultures (see results section), despite similar yield and morphology when harvests were possible (Figure S1A and B). We observed again, as for HSs, a senescent-like morphology with time in culture and gradual growth arrest in explants (Figure S1A) and CDCs (Figure S1C). On the contrary, Hyclone giFBS had a 100% successful culture rate, comparable harvest timing/frequency (Table S3), explant growth, CS yield (Figure S1), CDC proliferation rate, CDCs/IICSs gene expression profile and phenotype (Fig. 6). Overall, it was the best performing condition tested in our study, yielding completely satisfactory cell number and quality.

In conclusion, our data contribute to highlighting the issue of how the replacement of xenogenic media additives, such as FBS, is not always possible and highly cell-type dependent. For EU cGMP-complying isolation and production of CPCs in the form of CSs and CDCs, the combination of B27 supplement with Hyclone giFBS is the best performing protocol selected in our study. To our knowledge, this is the first comparative analysis aimed at optimizing culture conditions for the clinical translation of CPCs production under European cGMP standards.

## Supporting information

**Table S1** Sex, age and diagnosis of donor patients. **Table S2** Realtime PCR primers. **Table S3** Statistics of the timing for harvests from primary explant culture in tested AB human sera (HSs) and gamma-irradiated (giFBS) GMP-grade FBSs from different companies.

Figure S1 Primary explant cultures in 1 or 3% human AB serum.

Figure S2 Cell yield, morphology and proliferation of CPCs derived from gamma-irradiated Australian FBSs explant cultures.
