# Supplementary material for: Serum and supplement optimization for EU GMP-compliance in cardiospheres cell culture
Source: J Cell Mol Med. 2014 Jan 20;18(4):624–34. doi: 10.1111/jcmm.12210 (PMC4000114; doi:10.1111/jcmm.12210)
Supplement: Supplementary file 1 — Table S1 Sex, age and diagnosis of donor patients. Table S2 Realtime PCR primers. Table S3 Statistics of the timing for harvests from primary explant culture in tested AB human sera (HSs) and gamma-irradiated (giFBS) GMP-grade FBSs from different companies. [file jcmm0018-0624-sd1.doc]

Supplemental Table 1. Sex, age and diagnosis of donor patients.

|  | **SEX** | **AGE** | **DIAGNOSIS** |
| --- | --- | --- | --- |
| **1** | M | 55 | IC |
| **2** | F | 56 | IC |
| **3** | M | 63 | IC |
| **4** | M | 67 | IC |
| **5** | M | 42 | AI, MI |
| **6** | M | 67 | IC |
| **7** | M | 71 | IC |
| **8** | M | 56 | IC, MI |
| **9** | M | 49 | IC |
| **10** | F | 82 | IC, AS |
| **11** | M | 70 | IC |
| **12** | M | 70 | IC |
| **13** | M | 67 | IC |
| **14** | M | 62 | IC |
| **15** | F | 75 | IC |
| **16** | M | 58 | IC |
| **17** | M | 57 | IC |
| **18** | M | 65 | IC |
| **19** | M | 66 | IC |
| **IC** | ischaemic cardiomyopathy | | |
| **AI** | aortic insufficiency | | |
| **MI** | myocardial infarction | | |
| **AS** | aortic stenosis | | |

**Supplemental Table 2**. Realtime PCR primers.

| **Target** | **Sequence** | **T annealing** |
| --- | --- | --- |
| **GATA-4 fw** | GTTTTTTCCCCTTTGATTTTTGATC | 58 |
| **GATA-4 rv** | AACGACGGCAACAACGATAAT |  |
| **KDR fw** | AAAGGGTGGAGGTGACTGAG | 58 |
| **KDR rv** | CGGTAGAAGCACTTGTAGGC |  |
| **SMA fw** | ATGAAGATCCTGACTGAGCG | 58 |
| **SMA rv** | GCAGTGGCCATCTCATTTTC |  |
| **Nkx2.5 fw** | GGTGGAGCTGGAGAAGACAGA | 58 |
| **Nkx2.5 rv** | CGCCGCTCCAGTTCATAG |  |
| **TnI fw** | GGACAAGGTGGATGAAGAGA | 58 |
| **TnI rv** | AGGGTGGGCCGCTTAAACT |  |
| **GAPDH fw** | ACAGTCAGCCGCATCTTC | 58 |
| **GAPDH rv** | GCCCAATACGACCAAATCC |  |
| **Hsp90 fw** | CTGTGCCGTTGGTCCTGT | 58 |
| **Hsp90 rv** | CAATGACATCAACTGGGCAA |  |
| **Hsp70 fw** | ATGAGTATAGCGACCGCTGC | 58 |
| **Hsp70 rv** | TCCTTGGACTGTGTTCTTTGC |  |
| **Cx43 Fw** | AGGAGTTCAATCACTTGGCG | 56 |
| **Cx43 Rv** | GAGTTTGCCTAAGGCGCTC |  |
| **C-kit Fw** | GATGGATGGATGGTGGAGAC | 56 |
| **C-kit Rv** | GGGATTTTCTCTGCGTTCTG |  |
| **Thy-1 Fw**  **Thy-1 Rv** | CAGCGGAAGACCCCAGT  CGTTAGGCTGGTCACCTTCT | 58 |
| **MHC Fw**  **MHC Rv** | CAGAAGAAGAAGATGGATGC  CGCTGGTGTCCTGCTCCT | 58 |

**Supplemental Table 3. Statistics of the timing for harvests from primary explant culture in tested AB human sera (HSs) and gamma-irradiated (giFBS) GMP-grade FBSs from different companies. FBS in the second column refers to research-grade control serum from Lonza.**

|  |  | **Days for first harvest** | **Days between  harvests** | **Number of harvests** |
| --- | --- | --- | --- | --- |
| **HSs** | Lonza | 31.0±9.9 | 10.0±0.0 | 1.5±0.7 |
|  | Star | 26.0±2.8 | 13.5±4.9 | 2.0±0.0 |
|  | FBS | 24.0±0.0 | 7.0±2.1 | 4.0±1.4 |
|  |  |  |  |  |
| **giFBSs** | Lonza | 35.0±0.0 | 12.0±2.8 | 1.2±1.6 |
|  | Gibco | 39.7±8.1 | 12.7±2.3 | 1.8±1.6 |
|  | FBS | 37.8±6.3 | 11.6±2.3 | 3.4±0.5 |
|  |  |  |  |  |
|  | Hyclone | 32.7±4.0 | 8.7±1.5 | 3.7±0.6 |
|  | FBS | 32.7±4.0 | 8.7±1.5 | 3.7±0.6 |

Mean ± SD.

**Supplemental figure 1. Primary explant cultures in 1 or 3% human AB serum**. Representative bright field images of explant cultures in HS or FBS, showing no cell migration and growth compared to standard FBS. Scale bars=50µm.

**Supplemental figure 2. Cell yield, morphology and proliferation of CPCs derived from gamma-irradiated australian FBSs explant cultures.** Representative bright field images (a) of explant cultures and CSs from Lonza, Gibco and Hyclone giFBSs. CSs yield and dimension (b), expressed as percentage of effect versus standard FBS, were comparable in giFBSs cultures (when successful) compared to control FBS. CDCs from Lonza and Gibco giFBSs cultures had significantly lower proliferation efficiency than control FBS and Hyclone giFBS (c). Scale bars=50µm. *=P<0.001 Lonza and Gibco vs FBS.
