# Supplementary figures and images for: Serum and supplement optimization for EU GMP-compliance in cardiospheres cell culture
Source: J Cell Mol Med. 2014 Jan 20;18(4):624–34. doi: 10.1111/jcmm.12210 (PMC4000114; doi:10.1111/jcmm.12210)

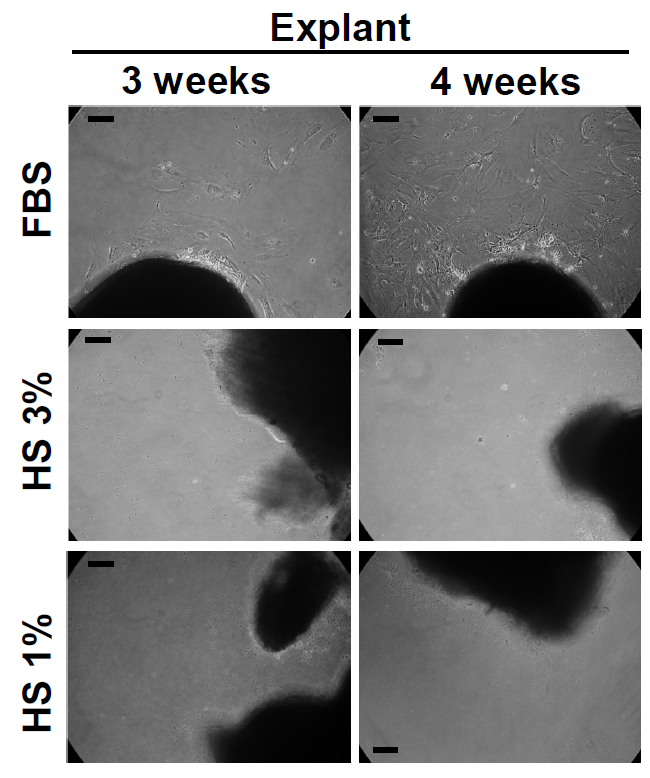

Supplement: Figure S1 [file jcmm0018-0624-sd2.tif]

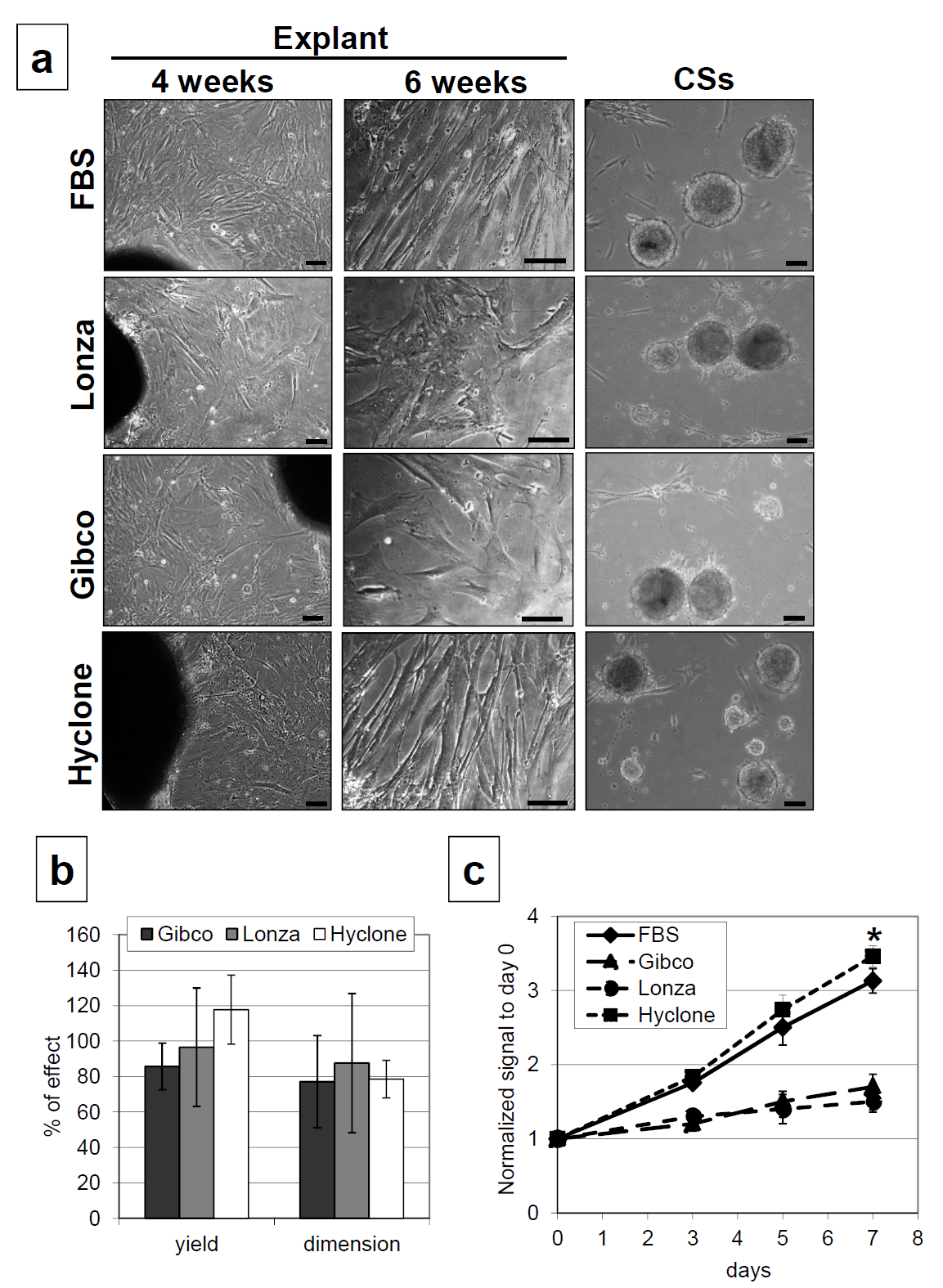

Supplement: Figure S2 [file jcmm0018-0624-sd3.tif]
